# The Price of the Induced Defense Against Pests: A Meta-Analysis

**DOI:** 10.3389/fpls.2020.615122

**Published:** 2021-01-21

**Authors:** Alejandro Garcia, Manuel Martinez, Isabel Diaz, M. Estrella Santamaria

**Affiliations:** ^1^Centro de Biotecnología y Genómica de Plantas, Universidad Politécnica de Madrid – Instituto Nacional de Investigación y Tecnología Agraria y Alimentación, Madrid, Spain; ^2^Departamento de Biotecnología-Biología Vegetal, Escuela Técnica Superior de Ingeniería Agronómica, Alimentaria y de Biosistemas, Universidad Politécnica de Madrid, Madrid, Spain

**Keywords:** fitness, growth, induced defenses, phytophagous, photosynthesis, plant, reproduction, trade-off

## Abstract

Plants and phytophagous arthropods have co-evolved for millions of years. During this long coexistence, plants have developed defense mechanisms including constitutive and inducible defenses. In an effort to survive upon herbivore attack, plants suffer a resource reallocation to facilitate the prioritization of defense toward growth. These rearrangements usually end up with a penalty in plant growth, development or reproduction directly linked to crop losses. Achieving the balance to maximize crop yield requires a fine tune regulation specific for each host-arthropod combination, which remains to be fully elucidated. The purpose of this work is to evaluate the effects of induced plant defenses produced upon pest feeding on plant fitness and surrogate parameters. The majority of the studies are focused on specific plant-pest interactions based on artificial herbivory damage or simulated defoliation on specific plant hosts. In this meta-analysis, the relevance of the variables mediating plant-pest interactions has been studied. The importance of plant and pest species, the infestation conditions (plant age, length/magnitude of infestation) and the parameters measured to estimate fitness (carbohydrate content, growth, photosynthesis and reproduction) in the final cost have been analyzed through a meta-analysis of 209 effects sizes from 46 different studies. Herbivore infestation reduced growth, photosynthesis and reproduction but not carbohydrate content. When focusing on the analyses of the variables modulating plant-pest interactions, new conclusions arise. Differences on the effect on plant growth and photosynthesis were observed among different feeding guilds or plant hosts, suggesting that these variables are key players in the final effects. Regarding the ontogenetic stage of a plant, negative effects were reported only in infestations during the vegetative stage of the plant, while no effect was observed during the reproductive stage. In addition, a direct relation was found between the durability and magnitude of the infestation, and the final negative effect on plant fitness. Among the parameters used to estimate the cost, growth and photosynthesis revealed more differences among subgroups than reproduction parameters. Altogether, this information on defense-growth trade-offs should be of great help for the scientific community to design pest management strategies reducing costs.

## Introduction

As plants are sessile organisms, they cannot escape from environmental cues, therefore, they have developed various mechanisms to overcome these biotic and abiotic stresses (Schoonhoven et al., 2005). During millions of years, the interaction between plants and their phytophagous opponents has shaped an intricate network of defenses and counter-defenses (Santamaria et al., 2013). Plant defenses can be classified broadly as constitutive (permanent) or induced (temporary) (Karban and Baldwin, 1997). Constitutive defenses are always present in the plant and do not depend on the attack of herbivores. These defenses are constantly activated but not always needed, which entails high costs for the plants (Karban, 2011). On the other hand, induced defenses are activated only in the presence of the attacker. In this context, the plant defense theory suggests that inducible resistance has evolved to reduce the costs of constitutive defenses (Heil and Baldwin, 2002; Cipollini et al., 2003; Zangerl, 2003; Cipollini and Heil, 2010). Although induced defenses allow plants to avoid the costs of implementing defenses in the absence of enemies, plants may suffer considerable damage during the time required to mount this defense response upon infestation (Frost et al., 2008). The implementation of plant defenses imposes a substantial demand for resources, which has been suggested to reduce growth. This negative impact on growth could result from diminished photosynthesis (Xia et al., 2009; Kirschbaum, 2011), which would decrease the overall pool of energy reserves, and/or from a diversion of resources away from growth and toward defense. As deficiencies in defense capabilities can result in plant damage, a balance between growth and defense must be achieved to optimize plant fitness (Huot et al., 2014). This growth-defense trade-off appears to result from plant allocation decisions intended to maintain optimal fitness while responding to a specific stress. Allocation costs can occur if large quantities of fitness-limiting resources are redirected to resistance traits. Such allocations might not be quickly recycled and hence are unavailable for fitness-relevant processes like growth or reproduction (Heil and Baldwin, 2002). It is well-known that the effects of induced defense on plant fitness depends on the specific pest and the target plant host but there are other variables very important in the final result that have been unexplored. Only Hawkes and Sullivan (2001) have compared the growth and reproduction costs in different plants (dicot, monocot, woody). However, the results are inconclusive, being the approach and data used quite restrictive because of the early date of publication and the limited number and type of available experiments. Several articles have shown a negative effect on plant fitness due to the induction of defenses against a specific pest, but the effect of the phytophagous specialization or their feeding guild have been poorly studied. In this sense, Nykänen and Koricheva (2004) did not find any significant effect of feeding specialization in the growth rate of woody plants, and Zvereva et al. (2010) performed a broader analysis but restricted to woody plants attacked by sap-feeders.

Most of the previous reports were focussed on one plant species infested with one particular pest under specific infestation conditions. Furthermore, each study uses different approaches to measure plant fitness which makes comparisons much more complicated. The proper measurement of fitness is critical to evaluate the duty paid for plant survival upon phytophagous infestation. The term “*fitness”* is related with “*reproductive success”* and during years the effects of herbivory on plant fitness were measured exclusively in terms of seed production (Strauss, 1997). Other parameters related with plant reproduction have also been used as fitness indicators, like seed yield, fruit production or seed size (Bardner, 1968; Sances et al., 1982; Summers and Newton, 1989; Bufon et al., 2020). However, the term “*fitness”* is more complex and the parameters used to estimate it have been changing along the time. As phytophagous feeding causes numerous alterations of the plant primary metabolism, several authors have monitored different parameters related to photosynthesis, transpiration, remobilization of carbon and nitrogen resources, sugar or water content as indicators of plant growth (Sances et al., 1979; Hutchison and Campbell, 1994; Watanabe and Kitagawa, 2000; Nykänen and Koricheva, 2004; Botha et al., 2006; Giri et al., 2006; Schmidt et al., 2009; Halitschke et al., 2011; Ochoa-Lopez et al., 2015; Machado et al., 2017; Santamaria et al., 2017, 2018; Bufon et al., 2020). In addition, as the leaves are the photosynthetic organs, relative growth rate (RGR), leaf number, leaf length, leaf area, specific leaf area, leaf/mass ratio, biomass or biomass allocation have been measured to evaluate plant growth (Vranjic and Ash, 1997; Nykänen and Koricheva, 2004; Schmidt et al., 2009; Sotelo et al., 2014; Ochoa-Lopez et al., 2015; Santamaria et al., 2018; Bufon et al., 2020). Although the most direct measure of fitness is to analyse the offspring of a plant, plant fitness has also been inferred from the study of the plant reproductive structures (flowers), propagules (seeds) or the actual reproductive success (number of germinating seeds) (Erb, 2018). However, because of the high costs and difficulties in the maintenance of an infested plant until the reproductive phase under controlled conditions, growth or photosynthetic parameters have been usually preferred to estimate plant fitness. In a recent study, Younginger et al. (2017) reviewed 170 datasets on plant fitness and discuss the metrics commonly employed for fitness estimations. They showed that biomass and growth rate are frequently used and often positively associated with fecundity, which in turn suggests greater overall fitness.

**Figure 1:**
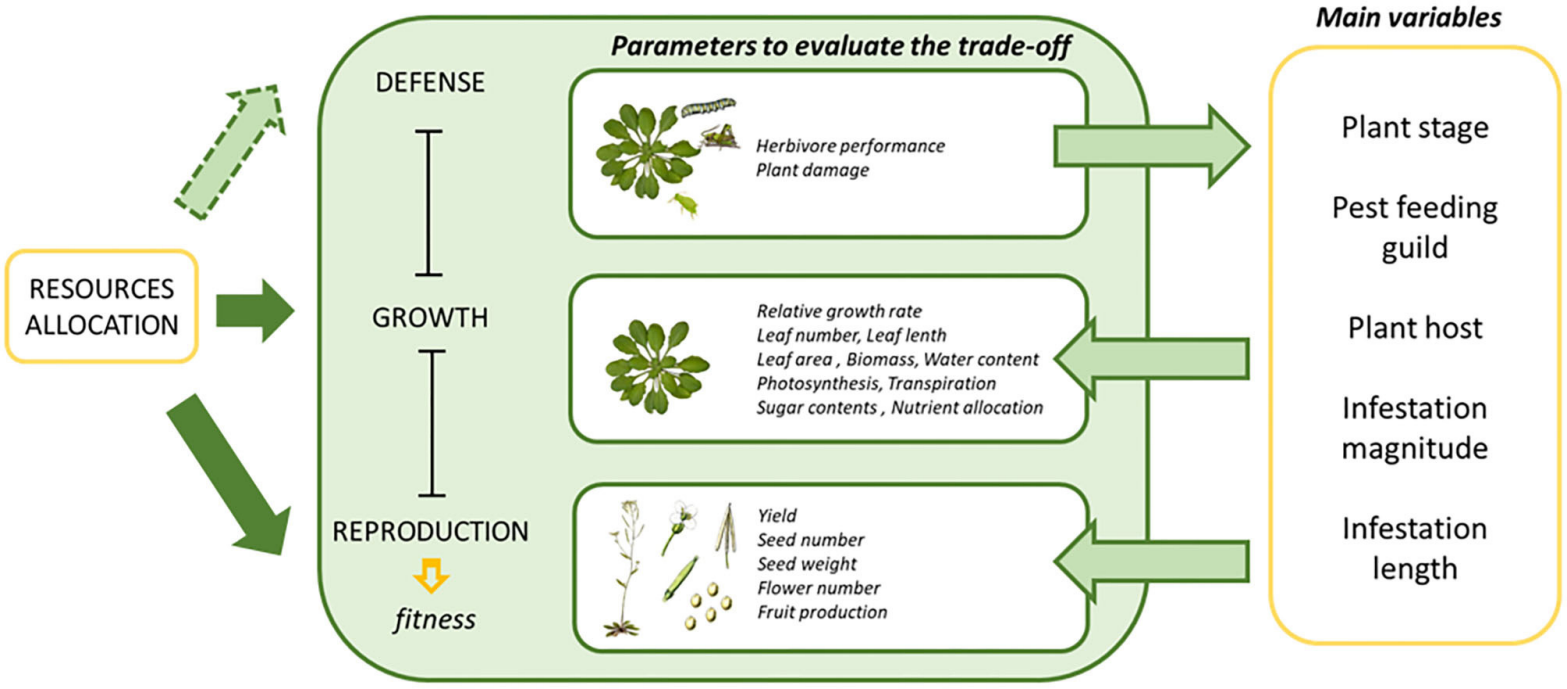
A diagram depicting the concept of growth defense tradeoffs, the parameters to evaluate the fitness and the main variables involved. Resource allocation is related to different processes by arrows. Solid arrows refer to natural processes occurring in plants, while arrows with dashed lines refer to events in which resource allocation is altered by herbivore infestation.

Many studies correlated growth rates and measures of defensive compounds with and without herbivore infestation (Paul-Victor et al., 2010; Züst et al., 2015). This approach could be enhanced by partitioning growth rates into physiological components much more directly related to nutrient allocation, like the net assimilation rate, activity of the photosystem or gas exchange (Rees et al., 2010; Li et al., 2016). Other variables that could have an effect of the final plant phenotype are the infestation conditions (magnitude and duration of the infestation) and the age of the plant when the infestation takes place. From these variables, only the effects of the plant ontogenetic stage have been previously studied in woody plants with simulated foliar damage (Nykänen and Koricheva, 2004). In this case, the growth of seedlings was reduced more than the growth of saplings. In addition, Zvereva et al. (2010) also evaluated similar variables, but the studies were also limited to woody plants and sap-feeding insects. Figure 1 summarizes the main parameters and variables used to estimate the trade-off between the physiological processes implicated in the allocation of resources upon plant herbivore infestation. In the present study we analyzed the effect of all these variables on plant fitness and surrogate parameters upon induction of plant defenses extracting general conclusions of the effects provoked by phytophagous herbivory.

## Materials and Methods

The Preferred Reporting Items for Systematic Reviews and Meta-Analyses (PRISMA; Moher et al., 2009) was applied to design a systematic review protocol to compile information on the following questions: (i) What are the effects of arthropod herbivory on plant growth, photosynthesis or reproduction?, (ii) Are these effects dependent on the parameters used to estimate plant fitness?, and (iii) How important are the variables mediating plant-pest interaction in the final effects?

### Compilation of the Database

A literature search was conducted to collect all relevant published data with no restriction of publication date related to the effect of arthropod herbivory on plant fitness. The publication screening process is provided in Supplementary Figure 1. Selection of the experiments was performed via online databases such as Google Scholar or ScienceDirect by a combination of keywords searches including “plant defense,” “herbivory attack,” “herbivory impact,” “plant fitness,” “plant insect herbivore interactions,” “tolerance,” “growth-defense trade-off,” “growth impact,” “photosynthesis impact,” “reproduction costs” or “fitness costs.” In this first step, a title and abstract screening procedure was followed, excluding those studies which do not contemplate outcomes related to fitness and surrogate parameters, studies with data related to other pathogens rather than herbivores, and studies with specified artificially defoliation treatments instead of herbivore attack, or with a lack of proper control data. Additional studies were also retrieved by examining the bibliographies of the selected papers. Once selected, to be included in the analysis, a study had to satisfy the following criteria: (1) Experiments include an arthropod herbivory treatment affecting plant growth, photosynthesis, reproduction or other parameters related to plant fitness; (2) The herbivory treatment has an appropriate control, not being altered their response by the application of herbicides, insecticides or previous herbivory damage; (3) If additional treatments were present in the experiment, data were selected from the control and herbivory treatment groups only; (4) The effects on plant fitness were measured immediately after herbivory removal with no long times after infestation measurements, not allowing the plants to recover from the stress; (5) The data required for the calculation of effect sizes (sample sizes, means, standard deviations, standard errors, *F-*test statistics or independent *t*-tests) can be extracted from the article in either numerical or graphical form; (6) The study provides information about both control and treated plants, including the study design and their characteristics. Measurements from different parameters, plant or arthropod species, plant stage, levels of infestation or experimental durations within a single study were considered to be distinct observations. Based on these criteria, experiments were excluded from the analysis if: (1) Fitness parameters were not included in the experimental set-up; (2) Herbivores included in the experiments were not arthropods but mammals or slugs; (3) Experiments were performed with artificial defoliation simulating herbivore damage instead of true direct phytophagous damage; (4) No control treatment was present in the experiment to compare the effect of the herbivore attack or control treatments were specified as plants with lower level of herbivory damage; (5) The effect of the fitness was measured weeks later after the herbivore feeding had taken place, being possible a recovery of the host plant; (6) Data available lack information for the extraction of the different effect sizes. In addition, one article was removed from the search because it was not possible to retrieve the full text of the paper.

### Variable Categorization

The retrieved studies reported measurements of plant responses that are directly related to plant fitness: plant growth, photosynthesis, reproduction and carbohydrate content. Plant fitness-related parameters were grouped for the analyses due to the low number of total studies retrieved. The final conformed groups and their individual variables in the database were: plant growth (leaf area, leaf length, number of leaves, plant height, plant biomass, relative growth rate (RGR), water content, and branch production), photosynthesis (photosynthetic rate, stomatal conductance, transpiration, pigment content, chlorophyll fluorescence, efficiency of PSII, and quantum yield), reproduction (days to first flower, number of flowers, flowering period, fruit production, fruit weight, fruit quality, pollen production and size, seed weight, seed production, yield), and carbohydrate content (glucose, fructose, sucrose, starch, sugar content).

We also collected several variables that might affect the plant responses to herbivory and could explain the differences in the plant fitness. These variables were: (1) Feeding guild. Arthropod species were classified into different groups based on their feeding mode. This classification includes chewing, phloem-feeding, cell-content feeder, gall-forming and leafminer insects. (2) Plant host. Plants were classified into crops, herbaceous and woody plants. The division between crops and herbaceous was performed according to the main use of the different species. Plants commonly used in crop management in fields in which production is usually quantified were termed as crops, while wild plants commonly found in nature were termed as herbaceous plants. (3) Plant stage. Based on the literature and the life cycle of each plant, plants were also classified based on their ontogenetic stage. The classification includes the early vegetative stage (from the seedling to the middle phase of vegetative stage), late vegetative stage (from the middle phase of the vegetative stage to the first reproductive event), and reproductive stage (after first reproductive event). (4) Infestation length. The time of infestation was categorized into short term and long term infestation time. Those experiments with an infestation length ranged between 0 and 10 days were classified as “short term,” while infestation lengths larger than 10 days were classified as “long term.” (5) Magnitude of infestation. Based on the type and amount of arthropod used in each experiment, division of the variable include light, medium or heavy infestation levels. This classification was based on the literature included in the experiment. In those papers where no specification of the infestation levels was included, classification was performed according to the information of related papers. A summary of the experiments with their effect size and classification in the different explanatory variables is provided in Supplementary Dataset 1.

### Data Extraction

The meta-analysis was conducted using R 4.0.0 (R Core Team, 2020) and RStudio 1.1.463 software (RStudio Team, 2016). Effect sizes were calculated as Hedge's *g*, the standardized mean difference (Gurevitch and Hedges, 2001) between the herbivore and control treatments by using the “esc” package (Lüdecke, 2018). Hedge's *g* is a similar measure than Cohen's *d* to calculate standardized mean differences, but it follows a different formula to calculate the pooled variance, controlling the slight bias in the small studies present in the Cohen's *d* (Hedges, 1981). If means and standard deviations or errors needed for the calculation of effect sizes were only present in graphs, the plugin “Figure Calibration” in ImageJ, available at: http://www.astro.physik.uni-goettingen.de/~hessman/ImageJ/Figure_Calibration/, was used to obtain data from plots (Hessman, 2009; Schneider et al., 2012). In some studies, means and standard deviations were unavailable. In those cases, univariate statistics such as *F-*test statistics or independent *t*-tests were transformed into Hedge's *g* estimates when present. When samples sizes were specified as a range of possible sizes, the lowest number was employed.

The individual measurements previously described to be related to plant fitness were extracted in agreement with the following rules. If more than one cultivar from the same plant species were analyzed in a single study, the most common host in nature for the herbivore was selected. If unspecified in the study, the susceptible one was selected over the resistant to estimate the real scope of the herbivory damage. However, in those cases where this resistance to the herbivore was not indicated, the data from all cultivars were included in the database, and aggregation of the effect into a single effect was calculated according to the “BHHR” procedure (Del Re, 2015). In those papers where varied length or magnitude of infestation were analyzed, one item per category (short/long term or light/medium/heavy) was selected. If a variable was measured in different tissues, foliage measurements were selected. In the case of photosynthesis, measurements including systemic responses were preferred over local responses as the data are more comparable to those studies where whole plant responses are analyzed. Finally, if the experiment was performed independently in two different years, data were selected randomly from one of them if the results were similar. When different, data was aggregated to include only one single effect. In the particular cases of the reproduction parameters of “days to first flower” and “flowering period,” a negative effect was considered when the time period increases. In these cases, longer periods to reach the first reproductive stage or longer flowering periods were considered to have a negative effect on reproduction.

All the analyses were performed following the random-effects model for pooling the different effect sizes using the “metagen” function of the “meta” package (Balduzzi et al., 2019). The random effects model assumes that, in addition to the sample error associated with each study, the true effect in each experiment will be influenced by several factors, including their characteristics, design and execution. Therefore, it is assumed that effects of individual studies deviate from the true intervention effect not only by sampling error, but also random variation. Assuming that in this meta-analysis sampling and random errors are likely to be important sources of variation, it was decided to follow this model. Once effect sizes were calculated, the experiments were divided into plant response parameters related to growth, photosynthesis, reproduction or carbohydrate content based on the individual variables analyzed. The magnitude of the treatment was considered to be statistically significant when the 95% confidence interval (CI) of the effect size did not overlap with 0 (Gurevitch and Hedges, 1993). Throughout the manuscript, the effect size and their confidence intervals as the mean effect size (Hedge's *g*) ± value to the 95% confidence interval limit (Hedge's *g* ± X.XX) was reported. In the cases where mean effect sizes were significantly different from 0, fail-safe number (*n*_*fs*_) was calculated using the weighted method of Rosenberg (2005). This number indicates the number of supplementary studies of null effect and mean weight needed to eliminate the significant effect. In addition, it was examined publication bias by performing the Egger's test of the intercept (Egger et al., 1997) for testing funnel plot asymmetry. Publication bias was considered if Egger's test was significant. Finally, Duval & Tweedie's trim-and-fill procedure (Duval and Tweedie, 2000) was followed in the cases where fail-safe number and Egger's test fail to reject the presence of publication bias. This method is also based on the funnel plot symmetry/asymmetry and is used to estimate the actual effect size that would be present in an asymmetric funnel plot by imputing “*missing”* studies until symmetry is reached. Egger's tests were conducted by using the “egger.test” function of the “dmetar” package (Harrer et al., 2019) while the trim-and-fill method was performed using the “trimfill” function of the “meta” package in R (Balduzzi et al., 2019).

In order to detect the presence of low-quality studies of small sample sizes, outlier detection was performed using the “dmetar” package (Harrer et al., 2019). Several potential outliers were identified in the data related to the general parameters of growth, photosynthesis and reproduction. Their effects on the results were tested by removing them from the data and re-running analyses. However, obtained results were very similar for analyses conducted with and without the potential outliers. For this reason, these studies were included in the final dataset. Subgroups of explanatory variables mediating plant-pest interactions were also analyzed individually to see if the presence of outliers altered their individual performance, showing high heterogeneity in each subgroup. Because of this reason, outliers on each subgroup were detected and removed to obtain more reliable effect sizes for the explanatory variables. To test whether effects on plant fitness in response to phytophagous differed among the explanatory variables discussed above (feeding guild, plant host, plant stage, infestation length and magnitude of infestation), studies were subdivided into corresponding groups, and between-group heterogeneity was examined using the χ^2^ statistic *Q*b (Gurevitch and Hedges, 2001).

### Statistical Analyses

A comparison of the mean effect sizes was performed to study the similarities and differences in the plant response among the subgroups present in the explanatory variables. Shapiro tests were conducted to check the presence of normality on the data. Levene's tests were used for assessing the presence of homogeneity of variance. When comparing two groups, statistical analyses were performed using the parametric Student's *t*-test with equal or unequal variance depending on the Levene's test results, and the non-parametric Mann-Whitney *U*-test for data with equal variance. If more than two groups were compared, normally distributed data were analyzed using One-way ANOVA. These analyses were followed by Bonferroni tests for unequal sample sizes, Dunnet T3 tests for unequal variances and sample sizes, and Kruskal Wallis tests for non-normally distributed data followed by Dunn's tests with Benjamini-Hochberg *p*-value adjustment. A significance threshold of 0.05 was applied in all tests. R version 4.0.0 was used for all analyses and generated plots.

## Results

### Meta-Analysis Data

Of the 1,255 papers initially identified, 1,210 come from searches in Google Scholar or ScienceDirect and 45 from the literature cited in these papers (Supplementary Figure 1). After duplicates were removed, 867 papers remained for abstract screening. Of these 867 studies, only 92 were finally identified as relevant articles to the review question. Finally, after carefully checking the preselected studies, a total number of 46 studies fitted our selection criteria. Of the excluded studies, 40% lacked information or showed low quality data for the extraction of effect sizes, 21% were studies performed with artificial defoliations instead of insects, 15% did not have an appropriate control to extract robust conclusions, 10% showed data measured weeks after infestation took place, 8% did not measure plant parameters related to fitness, and 6% were performed with no arthropod species. The 46 studies selected for the meta-analysis contained a total of 209 measurements of plant fitness responses to true direct herbivore damage, including observations on 44 plant species interacting with 46 arthropod herbivores (Supplementary Table 1). The most prevalent fitness parameters quantified were growth, photosynthesis and reproduction, with 62, 88, and 42 measurements, respectively. Carbohydrate content parameters were also included in the analyses, but with only 17 measurements. Within the growth parameters, 34 measurements were of plant biomass and relative growth rate, 18 of parameters related to the leaves like leaf number, leaf area or leaf size, and 10 of parameters related to plant height, stem size, branch production or water content. In the case of photosynthesis, measurements were more equally distributed in the quantification of different parameters. 26 out of 88 studies measured the efficiency of the PSII or the quantum yield, 24 the photosynthetic rate, 24 the stomatal conductance and transpiration, 11 the chlorophyll or pigment content, and 3 the CO_2_ assimilation and carbon exchange rate. Finally, the reproduction parameters conformed the most heterogeneous group, with 18 measurements of parameters related with seed production, like weight and yield, 13 related to fruit production and quality, 7 related to flowering or flower production, and 2 related to pollen production and size.

Parsing the selected studies allowed us to establish the following variables as partially explanatory of the variable plant responses: (1) Feeding guild (including chewing, cell-content, phloem-, gall-forming, and leafminer feeders), (2) plant host (including crops, herbaceous, and woody plants), (3) plant stage (including early and late vegetative and reproductive stages), (4) infestation length (including short term and long term infestation times), and (5) magnitude of infestation (including light, medium, and heavy densities of infestation). These variables led to the formation of different subgroups with or without equal responses to the growth, photosynthesis, reproduction and carbohydrate content effect sizes (Supplementary Dataset 1).

### Plant Infestation Exerts a Negative Effect on Plant Fitness When Growth, Photosynthesis, or Reproduction Parameters Are Measured

**Figure 2:**
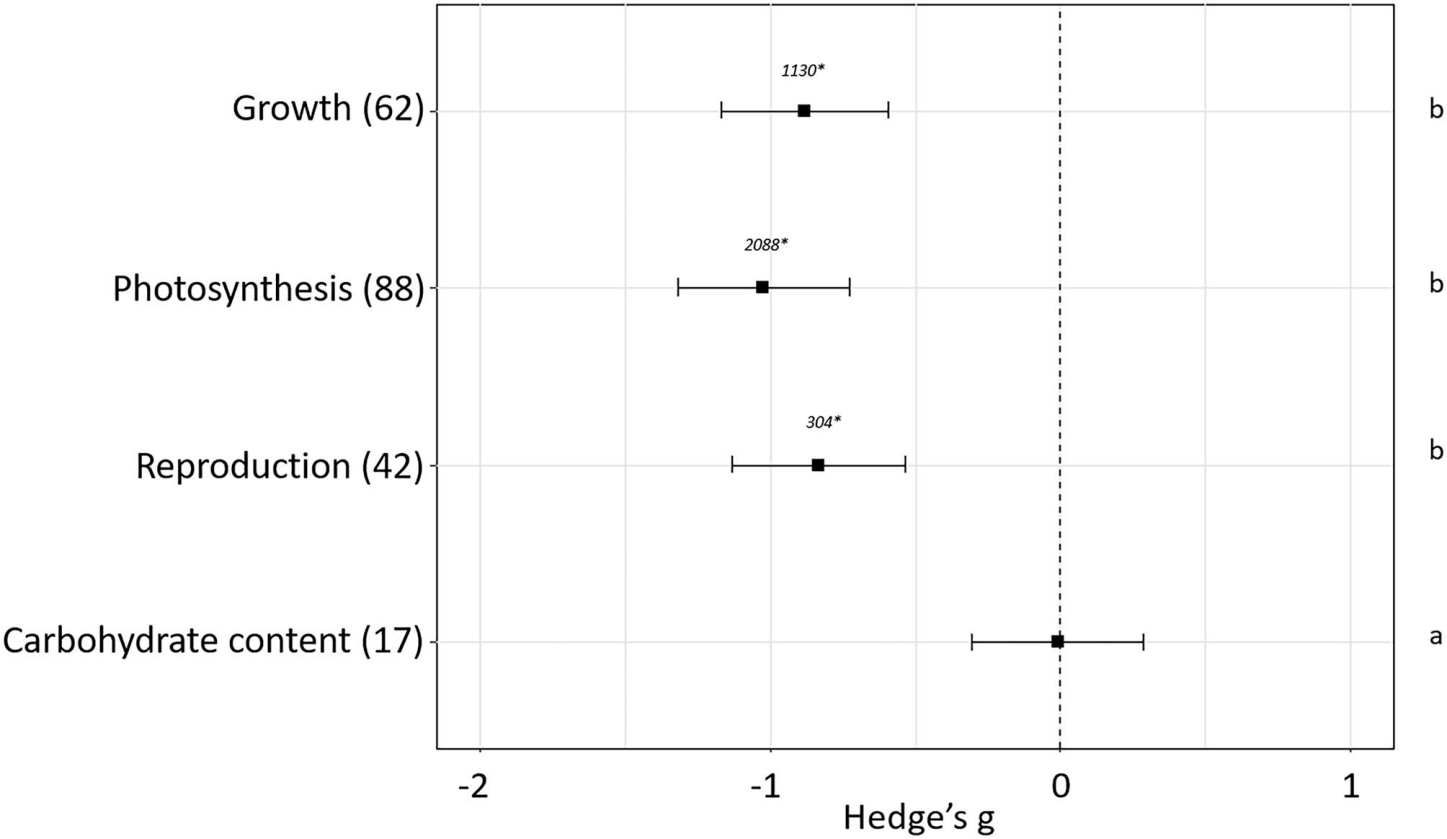
Effect sizes on general parameters related to fitness. Sample sizes are provided in brackets. Symbols specify mean values of Hedge's g with their 95%CI. Negative values indicate a higher negative effect in fitness on attacked plants than control plants. Different letters indicate significant differences between subgroups (*p*-value < 0.05). Statistical analysis were performed using Kruskal Wallis tests for non-normally distributed data followed by *post-hoc* Dunn's tests. Rosenberg's fail-safe numbers are reported in italics. An asterisk indicates a significant fail-safe number.

The effect of plant pest infestation in plant fitness has been evaluated. The experiments used in our meta-analysis showed differences in the effects depending on the sub-groups of parameters used to estimate plant fitness (*p* < 0.05). No effect sizes were detected when carbohydrate content (Hedge's g = −0.01 ± 0.29, *N* = 17) was measured as plant fitness indicator. However, negative effects were detected when growth (Hedge's g = −0.88± 0.29, *N* = 62), photosynthesis (Hedge's g = −1.029 ± 0.30, *N* = 88) or reproduction (Hedge's g = −0.83 ± 0.30, *N* = 42) parameters were measured (Figure 2). Besides the sub-group of parameters measured, different variables mediating plant-pest interactions as phytophagous feeding guild, plant host, plant stage at the moment of the infestation and length and magnitude of the infestation influenced final plant fitness. The impact of these variables on the fitness-related parameters is analyzed in the following sections.

### Effects of Plant-Pest Interaction Variables on Plant Growth

**Figure 3:**
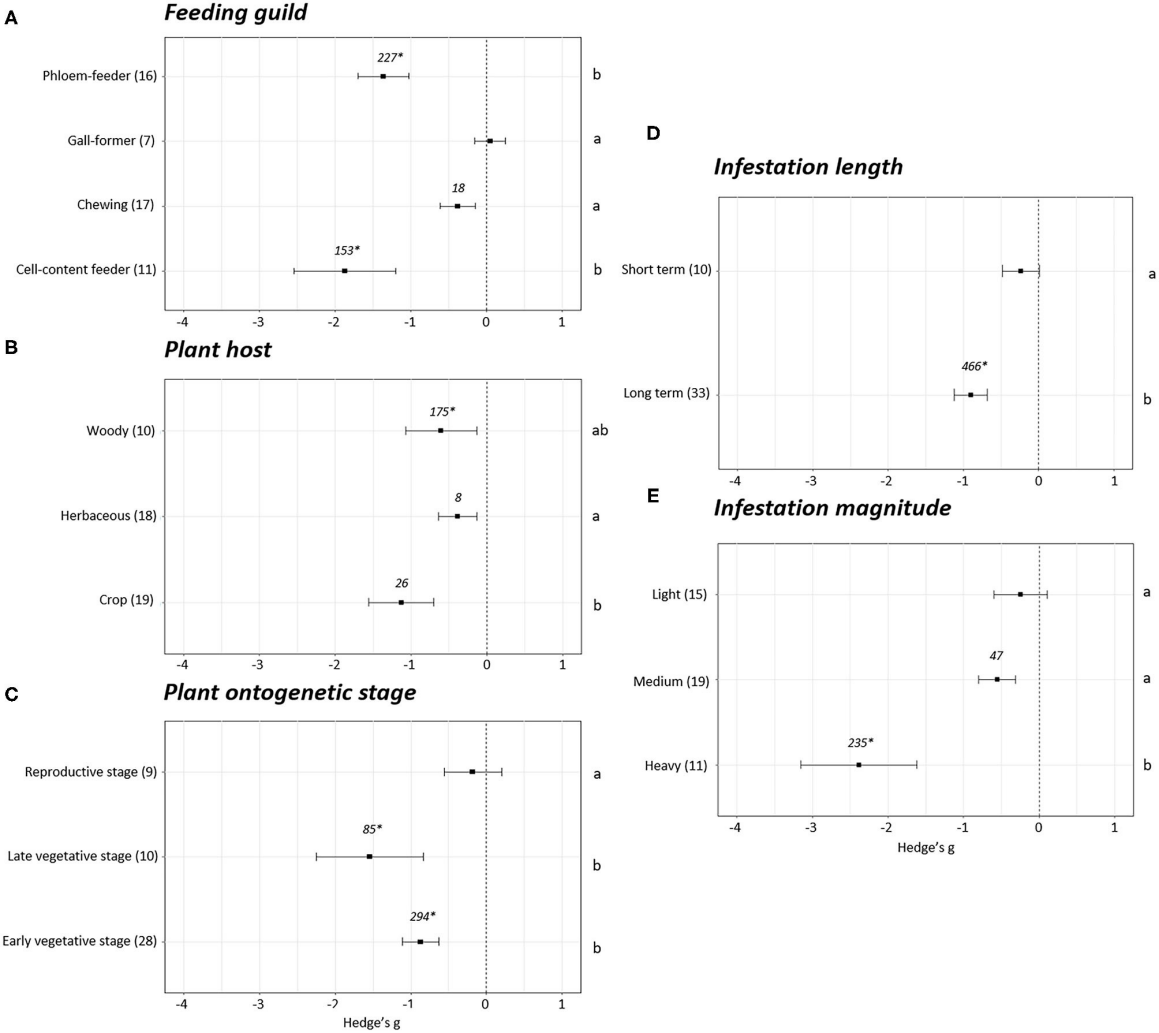
Growth effect sizes classified by subgroups. Fitness was analyzed based in different parameters related to growth. Subgroups included feeding guild **(A)**, type of plant host **(B)**, plant ontogenetic stage **(C)**, infestation length **(D)**, and magnitude of infestation **(E)**. Sample sizes are provided in brackets. Symbols specify mean values of Hedge's g with their 95%CI. Negative values indicate a higher negative effect in fitness on attacked plants than control plants. Different letters indicate significant differences between subgroups (*p*-value < 0.05). When comparing two groups, statistical analyses were performed using the parametric Student's *t*-test. If more than two groups were compared, statistical analysis were performed using One-way ANOVA for normally distributed data followed by Bonferroni test, and Kruskal Wallis tests for non-normally distributed data followed by *post-hoc* Dunn's tests. Rosenthal's fail-safe numbers are reported in italics. An asterisk indicates a significant fail-safe number.

Differences were observed when the plant growth effect sizes were studied attending to the feeding guild (*p* < 0.05). No effect sizes were detected in the experiments performed with gall-forming insects (Hedge's g = 0.05 ± 0.20, *N* = 7). However, negative effects on plant growth were revealed after infestation with cell content- (Hedge's g = −1.87 ± 0.67, *N* = 11), chewing (Hedge's g = −0.38 ± 0.24, *N* = 17), and phloem (Hedge's g = −1.36 ± 0.34, *N* = 16) feeders (Figure 3A). Plant growth was reduced to a greater extent in the experiments performed with cell-content and phloem feeders. Plant growth effect sizes were also analyzed depending of the plant host infested. The effects were always negative but significant differences were found between the effect observed in experiments performed on crops and herbaceous plants (*p* < 0.05). The most negative effects were detected when crops were infested (Hedge's g = −1.13 ± 0.43, *N* = 19) followed by woody (Hedge's g = −0.60 ± 0.47, *N* = 10) and herbaceous (Hedge's g = −0.38 ± 0.25, *N* = 18) plants (Figure 3B). Regarding the stage of the plants at the moment of infestation, no effects on plant growth were detected when the infestation was performed at the reproductive stage (Hedge's g = −0.18 ± 0.38, *N* = 9). Contrarily, negative effects were detected when the infestations were accomplished during the early (Hedge's g = −0.87 ± 0.24, *N* = 28) or late (Hedge's g = −1.54 ± 0.71, *N* = 10) vegetative stages (Figure 3C). The length and the magnitude of the infestation had also an impact on plant growth. Differences among effects were found by comparing short with long-term (Figure 3D) infestations and light/medium with heavy (Figure 3E) infestations (*p* < 0.05). While no effects on plant growth were detected for short-term infestations (Hedge's g = −0.24 ± 0.25, *N* = 10) and light infestation levels (Hedge's g = −0.24 ± 0.35, *N* = 15), negative effects were showed under long-term (Hedge's g = −0.90 ± 0.22, *N* = 33) and medium (Hedge's g = −0.55 ± 0.24, *N* = 19) and heavy infestations (Hedge's g = −2.38 ± 0.77, *N* = 11).

### Effects of Plant-Pest Interaction Variables on Photosynthesis

**Figure 4:**
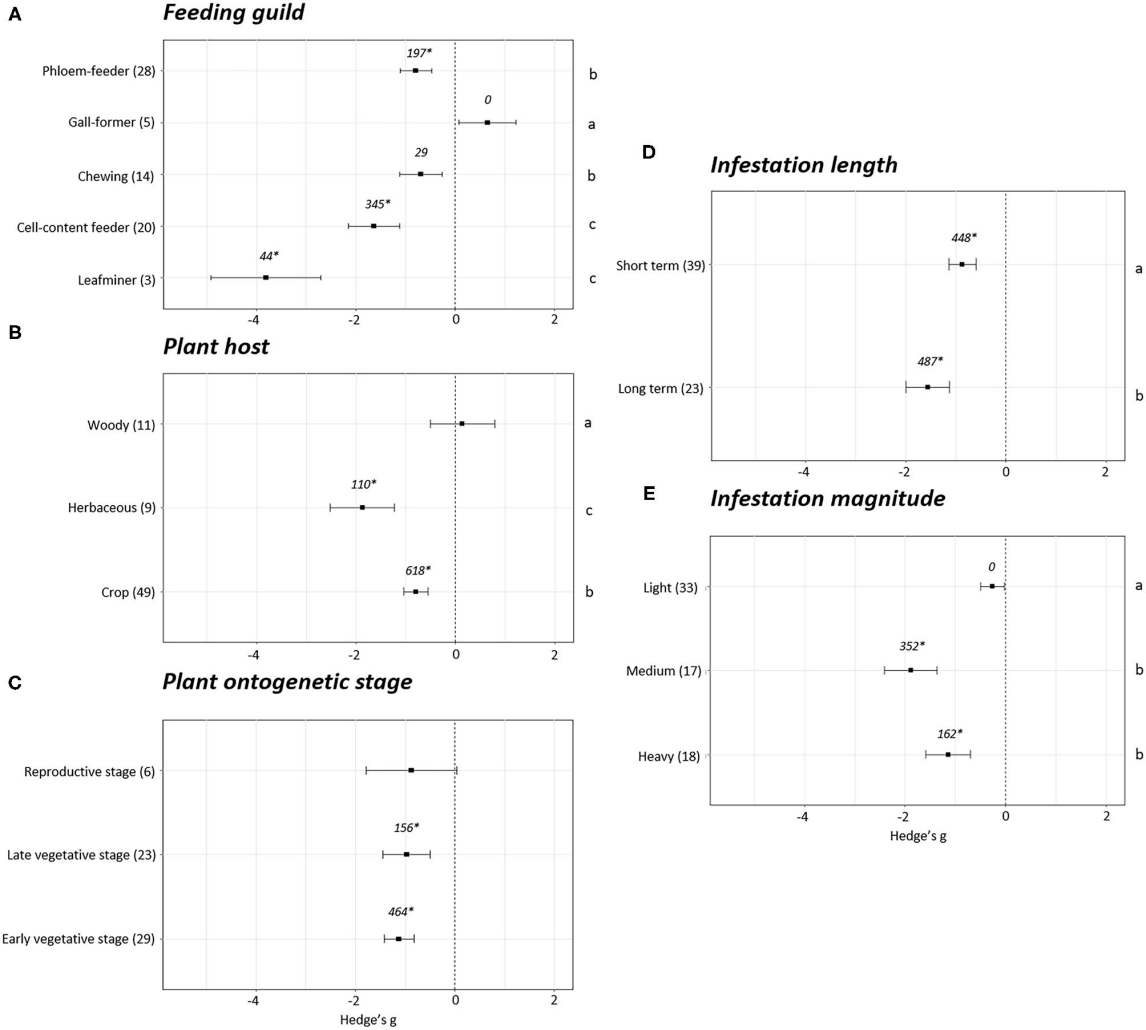
Photosynthesis effect sizes classified by subgroups. Fitness was analyzed based in different parameters related to photosynthesis. Subgroups included feeding guild **(A)**, type of plant host **(B)**, plant ontogenetic stage **(C)**, infestation length **(D)** and magnitude of infestation **(E)**. Sample sizes are provided in brackets. Symbols specify mean values of Hedge's g with their 95%CI. Negative values indicate a higher negative effect in fitness on attacked plants than control plants. Different letters indicate significant differences between subgroups (*p*-value < 0.05). When comparing two groups, statistical analyses were performed using the non-parametric Mann-Whitney *U*-test. If more than two groups were compared, statistical analysis were performed using One-way ANOVA for normally distributed data followed by Bonferroni test, and Kruskal Wallis tests for non-normally distributed data followed by *post-hoc* Dunn's tests. Rosenberg's fail-safe numbers are reported in italics. An asterisk indicates a significant fail-safe number.

Differences in plant fitness measured using photosynthesis parameters were found depending on the phytophagous way of feeding (*p* < 0.05). A positive impact on photosynthesis was detected only in the experiments performed with gall-forming arthropods (Hedge's g = 0.65 ± 0.58, *N* = 5). However, in this case, fail-safe number is equal to 0, indicating this effect cannot be distinguished from the null effect. The rest of groups showed negative effects on plant photosynthesis parameters (Figure 4A). Among them, the strongest negative effects were detected in plants infested with leafminers (Hedge's g = −3.81 ± 1.10, *N* = 3) and cell-content feeders (Hedge's g = −1.64 ± 0.52, *N* = 20) followed by chewing (Hedge's g = −0.69 ± 0.42, *N* = 14) and phloem (Hedge's g = −0.79 ± 0.41, *N* = 28) feeders. Regarding the plant host, no effects on photosynthesis were found in woody (Hedge's g = 0.14 ± 0.65, *N* = 11) plants while negative effects were detected in herbaceous plants (Hedge's g = −1.87 ± 0.64, *N* = 9) and crops (Hedge's g = −0.79 ± 0.24, *N* = 49) (Figure 4B). Statistical differences were found among the three groups of plants, being the most negative effects observed in the herbaceous plants followed by crops and woody plants (*p* < 0.05). Plant photosynthesis was unaffected when plants were infested at the reproductive stage (Hedge's g = −0.88 ± 0.91, *N* = 6). In contrast, clear negative impacts on photosynthesis were detected when plants were infested at early (Hedge's g = −1.13 ± 0.30, *N* = 29) or late vegetative stages (Hedge's g = −0.97 ± 0.48, *N* = 23) (Figure 4C). Negative effects on photosynthesis were also detected for short- (Hedge's g = −0.87 ± 0.27, *N* = 39) and long-term (Hedge's g = −1.56 ± 0.43, *N* = 23) infestations, showing a stronger negative (*p* < 0.05) in the long-term infestations (Figure 4D). According to the magnitude of the infestation, light infestations did not show negative effects on photosynthesis (Hedge's g = −0.26 ± 0.24, *N* = 33) while in medium (Hedge's g = −1.88 ± 0.52, *N* = 17) and heavy (Hedge's g = −1.14 ± 0.44, *N* = 18) infestations high negative effects were detected (Figure 4E). Statistical differences were found among light and medium/heavy infestations (*p* < 0.05).

### Effects of Plant-Pest Interaction Variables on Plant Reproduction

**Figure 5:**
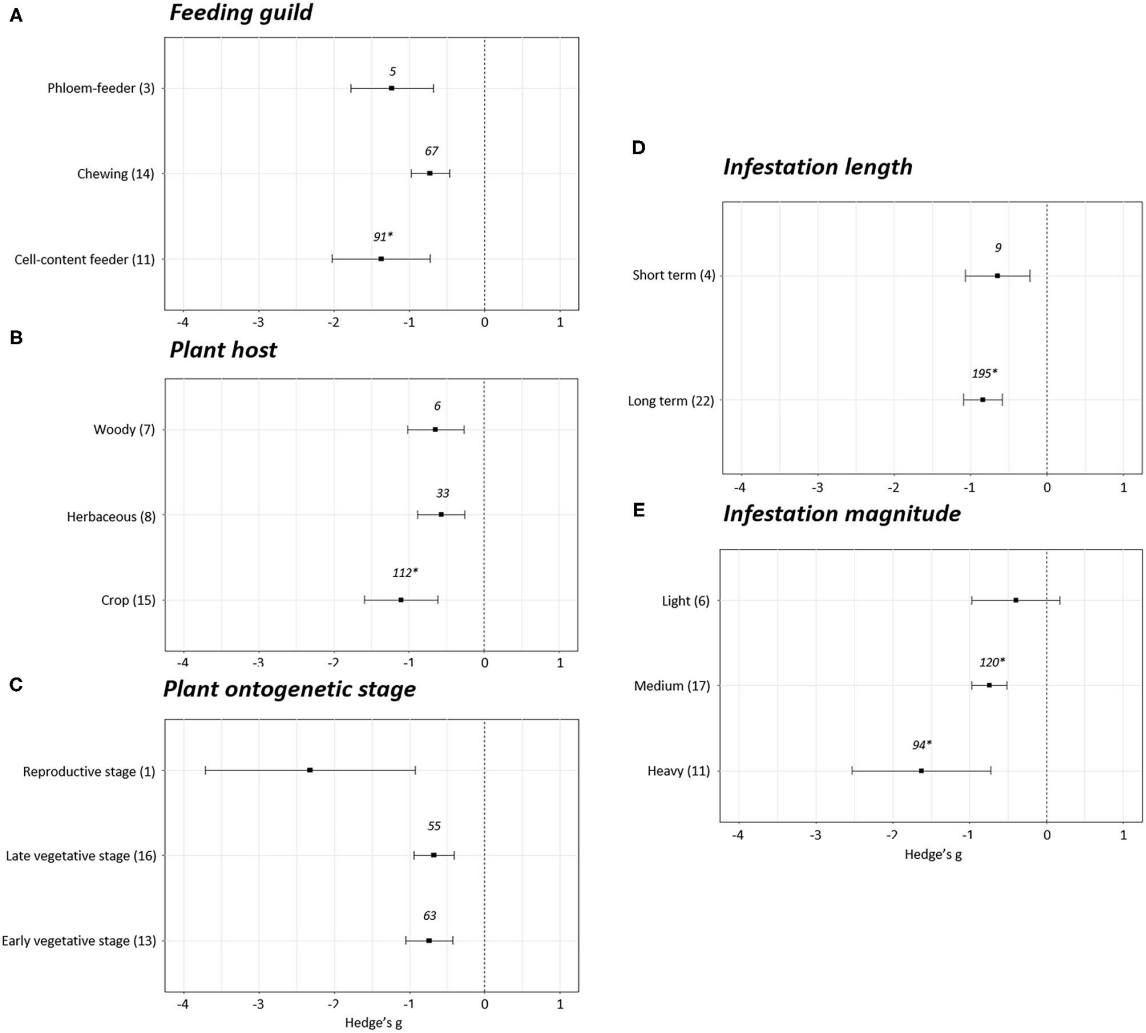
Reproduction effect sizes classified by subgroups. Fitness was analyzed based in different parameters related to reproduction. Subgroups included feeding guild **(A)**, type of plant host **(B)**, plant ontogenetic stage **(C)**, infestation length **(D)** and magnitude of infestation **(E)**. Sample sizes are provided in brackets. Symbols specify mean values of Hedge's g with their 95% CI. Negative values indicate a higher negative effect in fitness on attacked plants than control plants. No statistical differences are present between subgroups (*p*-value > 0.05). When comparing two groups, statistical analyses were performed using the parametric Student's *t*-test with Welch's correction for unequal variances. If more than two groups were compared, statistical analysis were performed using One-way ANOVA for normally distributed data. Rosenberg's fail-safe numbers are reported in italics. An asterisk indicates a significant fail-safe number.

Most variables reported negative effect sizes when reproduction was altered by phytophagous infestation (Figure 5). In the case of the feeding guild, chewing (Hedge's g = −0.72 ± 0.25, *N* = 14), phloem- (Hedge's g = −1.23 ± 0.54, *N* = 3) and cell- content feeders (Hedge's g = −1.38 ± 0.65, *N* = 11) displayed a negative effect on plant reproduction (Figure 5A). Attending to the plant host infested, a negative impact was found independently of the plant host of study. Statistical differences among crops (Hedge's g = −1.10 ± 0.49, *N* = 15), herbaceous (Hedge's g = −0.57± 0.32, *N* = 8) and woody plants (Hedge's g = −0.64 ± 0.37, *N* = 7) were not found (Figure 5B). An effect on reproduction was also observed when plant stage and infestation length were studied. Negative effects on reproduction are found at early vegetative (Hedge's g = −0.74 ± 0.31, *N* = 13), late vegetative (Hedge's g = −0.68 ± 0.27, *N* = 16) and reproductive stages (Hedge's g = −2.32 ± 1.39, *N* = 1), as well as in short-term (Hedge's g = −0.65 ± 0.42, *N* = 4) and long-term (Hedge's g = −0.84 ± 0.25, *N* = 22) infestations, but no statistical differences were extracted among the subgroups within each variable (Figures 5C,D). Reproductive stage data cannot be used to infer effect sizes as composed by a unique experiment. Finally, no effects on reproduction were identified in experiments performed with light infestations (Hedge's g = −0.40 ± 0.58, *N* = 6), but negative effects were detected in medium (Hedge's g = −0.74 ± 0.23, *N* = 17) and heavy (Hedge's g = −1.63 ± 0.90, *N* = 11) infestations (Figure 5D).

### Assessment of Risk Bias

Publication bias represents a major concern to consider when performing a meta-analysis. Significant effects are more likely to be published than non-significant effects (Borenstein et al., 2011), leading to a probable scenario of overestimation of effects. Therefore, the quality of the data of the final database is of critical importance to extract robust conclusions.

In our case, the risk of including overestimating effects in our final database was analyzed by calculating Rosenberg's fail-safe numbers and performing Egger's tests. Publication bias was suggested when Egger's tests were significant. However, it was safely ignored when the fail-safe numbers were >5*n* + 10, where *n* represents the number of studies, which are considered robust values against publication bias (Rosenthal, 1991; Rosenberg, 2005). In the cases where sample size was small (lower than 10), Egger's test may lack the statistical power to detect bias and consequently, *p*-values were not considered. In our data, fail-safe numbers and Egger's test allowed to reject the presence of publication bias in most cases, suggesting a non-overestimation of the effects. When neither Egger's test nor fail-safe number were able to reject the risk of publication bias, Duval & Tweedie's trim-and-fill procedure was performed, being effect sizes recalculated for estimating the true effect size without the presence of publication bias (Duval and Tweedie, 2000). Fail-safe numbers, Egger's test values and groups where effect sizes were recalculated by the trim-and-fill method are provided in Supplementary Table 2.

## Discussion

### Pest Infestation Produces an Overall Impact on Plant Fitness

Plant induced defenses are assumed to be energetically costly and have an impact on plant fitness. Fitness is a quite complex concept that has been traditionally evaluated by measuring growth, photosynthesis, carbohydrates or reproduction parameters (Züst and Agrawal, 2017). Our meta-analysis supports this assumption and shows a clear negative impact of pest infestation on plant growth, photosynthesis and reproduction rates. This negative impact was independent of several variables mediating plant-pest interactions critical for the final plant phenotype, such as the effect of the arthropod feeding guild, plant host, plant stage and the length and magnitude of the infestation. However, differences among the negative effects produced by the subgroups analyzed on each variable are expected to be found. In contrast, when carbohydrate content was measured, no effects were detected. This could be due to the more limited number of experiments found for carbohydrate content together with a variable effect either slightly positive or negative on the final plant fitness.

### Effect of Feeding Guild on Plant Fitness

Phloem and cell content feeders inflict less mechanical damage than chewing, mining or gall forming phytophagous because only their stylets penetrate in the sap or the mesophyll/epidermal cells (Wondafrash et al., 2013). Among the data assembled on this meta-analysis, most of the experiments were performed with phloem-, cell content- and chewing insects. Independently of the parameter measured, the infestation with these arthropods had a negative impact on plant fitness. Interestingly, our meta-analysis indicates that the impact on plant growth was higher after infestations with phloem or cell content feeders than upon chewing insects. This result lines with Zvereva et al. (2010) who observed that the effects on plant photosynthesis were negatively stronger upon sap-feeder infestation than when plants were treated with chewing insects. This result could be explained by the mechanical consequences of the different feeding modes and the parameters measured to estimate fitness. Chewing insects produce a loss of the attacked tissue while no tissue removal is present after phloem- or cell-content feeder attack. As a consequence, the remaining amount of damaged tissue is higher after phloem- and cell-content infestation than after chewing insect attack. However, lower amounts of plant tissue due to chewing infestations were not correlated to lower plant biomass measurements when comparing to phloem- and cell-content infestations. This result may be associated to the different interactions with plant signaling pathways described for insect species differing in the feeding style.

The central phytohormones that mediate between signal recognition and activation of defenses against pests are Jasmonic Acid (JA) and Salicylic Acid (SA). Whereas JA regulates the induced defenses against chewing insects (Schmiesing et al., 2016) and mesophyll sucking mites (Zhurov et al., 2014; Alba et al., 2015; Martel et al., 2015), SA-regulated responses are induced by phloem-feeding insects (Kawazu et al., 2012; Thaler et al., 2012) and also by mesophyll sucking mites (Kant et al., 2004; Santamaria et al., 2017, 2019). Once plant defense responses are activated at the site of infestation, a systemic defense response is triggered to protect distal undamaged tissues, generating a long-lasting induced resistance (Durrant and Dong, 2004). There are two forms of induced resistance: systemic acquired resistance (SAR) and induced systemic resistance (ISR). The establishment of SAR is associated with increased levels of SA (Mishina and Zeier, 2007; Tsuda et al., 2008). In fact, mutant and transgenic plants impaired in SA signaling are incapable of developing SAR, reflecting the critical role of SA in the SAR signaling pathway (Durrant and Dong, 2004). On the other hand, ISR is a SA-independent pathway dependent on JA and ethylene (ET) signaling (Choudhary et al., 2007). Therefore, it is suggested a higher SAR systemic response of the plant led by different regulation of JA and higher expression of SA when phloem- and cell-content feeders are present. This systemic response would produce an allocation of the fitness resources to invest in defense, reducing the growth of the plant in a manner much more severe than the one elicited by the chewing insects, which would produce a more reduced systemic response. In the case of photosynthesis, the lowest negative effects found in chewing insects are easily understood as photosynthesis parameters are measured in the remaining tissue, which is more negatively affected in the case of cell-content feeders. Regarding leafminers, only three experiments were found and all of them measured photosynthesis to study the infestation impact on plant fitness. The effect of leafminer feeding on photosynthesis was the one with the strongest negative effect found among the different feeding guilds. According to this, it has been previously suggested that, in response to leafminers, plants try to minimize losses via trade-offs between the negative impact on photosynthesis and the positive effects on water use efficiency (Pincebourde et al., 2006). When plants were infested with gall forming insects, the effect on plant fitness varied depending of the parameters measured. While no effects were detected on growth parameters, a null or even a slightly positive impact was present on photosynthesis. Aldea et al. (2006) detected negative effects on plant photosynthesis upon gall forming infestation when quantum yield was measured solely in the damaged patches. This result could be due to the feeding way of these insects, which induced the development of pathologically isolated cells where the photosynthesis decreases (Huang et al., 2015). However, when photosynthesis was measured in the whole plant, the effects of the infestation were slightly positive, suggesting a compensatory response to the damage generated in the galls (Aldea et al., 2006).

The specificity of induced plant responses has been previously associated to the recognition of specific feeding styles and damage patterns and/or herbivore specific elicitors in salivary secretions and regurgitates introduced in the plant during the feeding process (Santamaria et al., 2018). These responses will turn out with very specific allocation resources that will have a positive, negative or neutral impact on plant fitness. Our results strongly indicate that the induction of plant defenses by herbivorous arthropods causes adverse effects on plant growth and photosynthesis, which severity depends on the feeding guild of the phytophagous species.

### Effect of Host Plant on Plant Fitness

Our meta-analysis showed that the cost of the induced defenses varied depending if the plant host is a woody, an herbaceous or a crop species. When growth, photosynthesis and reproduction parameters were measured, the effects of the infestation on plant fitness were always negative independently of the plant host, with the exception of a null or slightly positive effect detected for woody plants. Similar results were found in a previous meta-analysis performed with woody plants upon natural or simulated feeding in which the photosynthesis increment was justified by the elevated sink demands in plants recovering from damage (Nykänen and Koricheva, 2004). It has also been suggested that partial defoliations on *Eucalyputs globulus* increase the photosynthetic rates due to an increase of the maximum rate of carboxylation and RuBP regeneration (Turnbull et al., 2007). Additional findings support the importance of the plant host in the cost of induced defenses by pests. In the above described meta-analysis on woody plants, the growth rate of evergreen plants was reduced more than in deciduous plants (Nykänen and Koricheva, 2004). However, deciduous and evergreen woody plants did not differ in their abilities to tolerate damage imposed by sap-feeders (Zvereva et al., 2010). A higher negative effect of the herbivory was reported on monocots than on dicots or woody plants (Hawkes and Sullivan, 2001). Furthermore, Bownes et al. (2010) suggested that if the hosts included in the analysis were limited to real hosts infested in the field the result should be more representative and marked.

### Effect of Plant Stage on Plant Fitness

The structures associated with plant growth, defense and reproduction require a complex set of resources, including minerals like carbon, nitrogen and phosphorus (Bazzaz et al., 1987). Variations in the allocation of these resources occur through differences in the chemical composition of plant structures, the relative mass of the structures or organs, and the relative numbers of the structures produced by a plant. Our meta-analysis showed that when plants were infested in early or late vegetative stages, the reallocation of resources ends up with negative effects on plant growth, photosynthesis and reproduction. However, when the infestations were carried out during the reproductive stage, this resource reallocation does not ended up with an impact on plant fitness. Our hypothesis is that when the reproductive stage is reached, the plant prioritizes growth against defense to guarantee a proper development of seeds. According to this, Rusman et al. (2019) have observed that the negative consequences of herbivory on flowering traits and reproductive output were stronger when plants were attacked early in life that when plants have already ensured the reproductive stage.

### Effect of Length and Magnitude of the Infestation on Plant Fitness

As expected, our meta-analysis showed negative effects on all the parameters measured to estimate fitness when long-term infestations were analyzed. In the short-term infestations, a negative impact was detected on photosynthesis and reproduction, but not on growth, and always with lower effect sizes than in long-term infestations. These results suggest that longer exposures of plants to a stress lead to larger negative impacts on fitness probably due to a longer time of investing in plant defenses rather than in growth. If more extended periods of infestation lead to higher population densities, larger negative effects are expected to take place in the plant due to a higher number of individuals feeding on it. In fact, our meta-analysis indicates the strong importance of the level of the infestation in the final plant fitness. The experiments performed with medium or heavy infestation levels produced a final negative impact on plant fitness independently of the parameter measured, but when the infestation level was light no effects were detected. According to these results, light density of infestations could allow plants to recover successfully from the initial stress. Thus, light densities of pests remaining and surviving in the plant could lead to a reproductive success of both the plant and the arthropod species.

### Differences Among Sub-groups Within Each Variable Depends on the Parameters Used to Estimate Plant Fitness

Significant differences in the effect sizes among sub-groups within each variable were found when growth and photosynthesis parameters were evaluated. However, when reproduction parameters were analyzed, no significant differences were detected within any of the variables. This absence of statistical differences could be associated to the lowest number of experiments that used reproduction parameters to estimate plant fitness, probably due to the complex and tedious work of estimating reproduction parameters. It could be possible that data collected for reproductive parameters come only from experiments in which plants are able to survive until the reproductive phase. Those experiments in which plant prematurely die due to the biotic stress were not likely to be used for analyzing reproductive parameters, and therefore, not being reflected this negative effect in the final analyses. Another possibility is that the parameters used to quantify reproduction could be introducing additional variability. For example, the number of flowers can be used to estimate the reproductive potential of a plant. However, the quantification of the number of flowers did not lead to corresponding effect sizes in seed production when herbivores are affecting pollination (Herrera et al., 2002) or when flowers are shed prematurely (Niesenbaum, 1996). In this case, the number of seeds could lead to higher or lower effect sizes than the number of flowers. More experiments are required to know if the negative effects found in reproduction without significant differences within variables are due to the limited number of experiments compiled, to the intrinsic variability of the methods to measure reproduction or to a physiological reason based on a plant specific response.

## Conclusions

The cost of inducible defenses in plant fitness has been traditionally focused on the impact on a specific plant-pest system under its optimal experimental conditions. This meta-analysis was designed to obtain a broad view of defense-growth trade-offs considering the most important parameters to estimate fitness and the main variables mediating plant-pest interactions. In fact, it is the first meta-analysis not focused only on a specific plant host and using data coming from experiments with direct feeding damage not artificially simulated. Our results enable us to extract some reliable conclusions: (i) The effects observed on plant growth, photosynthesis and reproduction upon plant-pest interaction are negative independently of the variables mediating plant-pest interphase; (ii) Due to the limited number of studies or the dependence on the specificity of the response and the variables modulating plant-pest interaction, herbivore infestations do not show a significant effect on carbohydrate content of plants; (iii) The feeding guild of the arthropods and the plant host used are definitely decisive in the final taxes that the plant pay for defense; (iv) The ontogenetic stage of the plant when the infestation takes place, the durability, and the density of the infestation are key factors in the final fitness phenotypes, independently of the parameter used to estimate costs; (v) Differences among subgroups within each variable depends on the parameters used to estimate plant fitness, being growth and photosynthesis the best to discriminate the impact on them.

Globally, the meta-analyses presented here convincingly shows that induced defenses have a fitness cost with a relevance that varies according to the parameter used to estimate it and an impact that depends on the variables mediating the particular plant-pest interaction. Increasing our knowledge about pest impact on plant fitness, understanding the importance of the variables mediating plant-pest interactions and identifying the proper parameters to estimate plant fitness would be key for the proper design of experiments focused on deciphering the mechanisms under the trade-off established upon plant-pest interactions. In this next step, these mechanisms and the particularities behind these trade-offs established upon different plant-pest specific combinations will be unveiled and used in the design of specific pest management strategies. These programs will be focused on the production of more resistant plants minimizing plant fitness costs, allowing an improvement of the current production systems, which will be very important in the current context of increasing demands and costs, linked to the constant climate change that our agricultural systems are facing nowadays.

## Data Availability Statement

The original contributions presented in the study are included in the article/Supplementary Materials, further inquiries can be directed to the corresponding author.

## Author Contributions

MES conceived the idea. AG performed most of the experiments. MES and AG wrote the original draft of the manuscript. MM and ID made substantial contributions to enhance a final version of the manuscript. All authors have read and agreed to the published version of the manuscript.

## Conflict of Interest

The authors declare that the research was conducted in the absence of any commercial or financial relationships that could be construed as a potential conflict of interest.
