# Supplementary material for: The Price of the Induced Defense Against Pests: A Meta-Analysis
Source: Front Plant Sci. 2021 Jan 21;11:615122. doi: 10.3389/fpls.2020.615122 (PMC7859116; doi:10.3389/fpls.2020.615122)
Supplement: Supplementary file 1 [file Data_Sheet_1.doc]

**
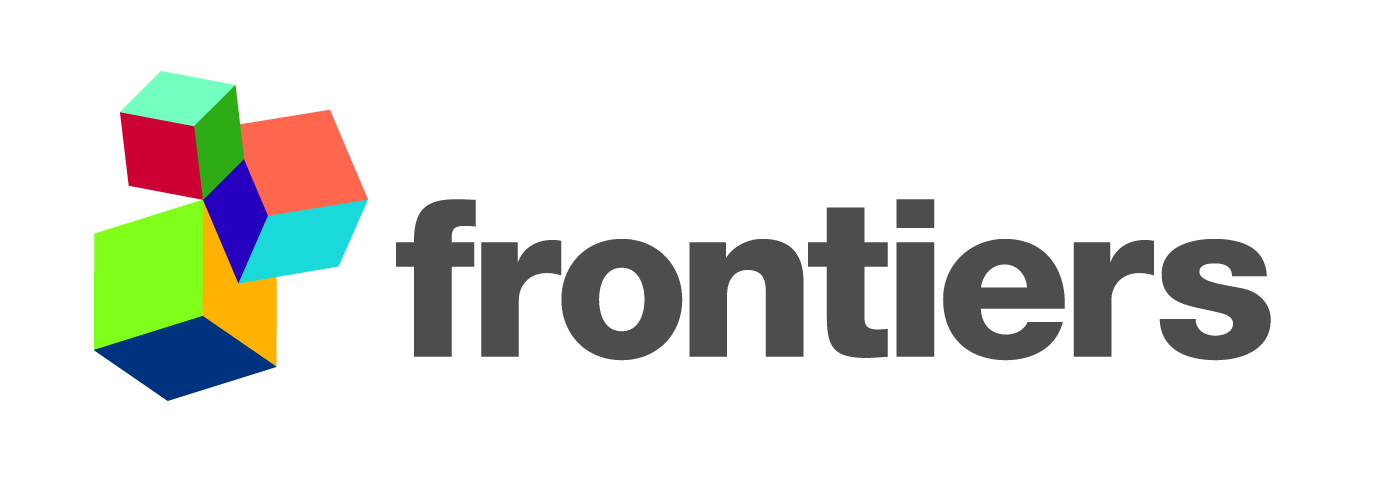
**

Supplementary Material

**The Price of the Induced Defence Against Pests:**

**a Meta-Analysis**

Alejandro Garcia, Manuel Martinez, Isabel Diaz, M. Estrella Santamaria^*^

*** Correspondence:** M. Estrella Santamaria: [me.santamaria@upm.es](mailto:me.santamaria@upm.es)


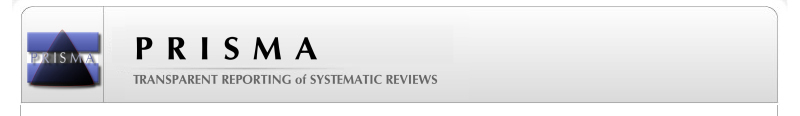
**PRISMA 2009 Flow Diagram**

**Screening**

**Included**

**Eligibility**

**Identification**

Records identified through database searching
(n =1210)

Additional records identified through other sources
(n =45)

Records after duplicates removed
(n = 867)

Records screened
(n = 867)

Records excluded
(n =775)

Full-text articles assessed for eligibility
(n = 92)

Full-text articles excluded, with reasons
(n = 46)

Studies included in qualitative synthesis
(n = 46)

Studies included in quantitative synthesis (meta-analysis)
(n = 46)

##

*From:*  Moher D, Liberati A, Tetzlaff J, Altman DG, The PRISMA Group (2009). *P*referred *R*eporting *I*tems for *S*ystematic Reviews and *M*eta-*A*nalyses:

The PRISMA Statement. PLoS Med 6(7): e1000097. doi:10.1371/journal.pmed1000097

**For more information, visit** [**www.prisma-statement.org**](http://www.consort-statement.org/)**.**

**Supplementary Figure 1.** PRISMA flow diagram detailing screening process of articles included in analysis.

**Supplementary Table 1.** Reference list for studies included in the meta-analysis.

**Nº Publications**

1. Agrawal, A. A., Strauss, S. Y., and Stout, M. J. (1999). Costs of induced responses and tolerance to herbivory in male and female fitness components of wild radish. *Evolution* 53:1093-1104. doi:[10.2307/2640814](https://www.researchgate.net/deref/http%3A%2F%2Fdx.doi.org%2F10.2307%2F2640814?_sg%5B0%5D=U1KOJRs0rezwjSVB9I8ZtHuKxtH3uBvJJvldwJ_KEI1ZHgLhgPNBTn01PbjuYAUtt3e0gnoasoGoyxrFgunyI_Lnwg.y4zoz3RoWyh48-TZuODr5fhKcz1iieoCUkKItGp_E4Ny-N_huftvItaZva1ZINzAMK4XAHipI3chYfKK4LmHlg)
2. Aldea, M., Hamilton, J. G., Resti, J. P., Zangerl, A. R., Berenbaum, M. R., Frank, T. D., and DeLucia, E. H. (2006). Comparison of photosynthetic damage from arthropod herbivory and pathogen infection in understory hardwood saplings. *Oecologia* 149:221-232. doi:10.1007/s00442-006-0444-x
3. Bardner, R. (1968). Wheat bulb fly, *Leptohylemyia coarctata* Fall., and its effect on the growth and yield of wheat. *Ann. Appl. Biol*. 61:1-11. doi:[10.1111/j.1744-7348.1968.tb04504.x](https://www.researchgate.net/deref/http%3A%2F%2Fdx.doi.org%2F10.1111%2Fj.1744-7348.1968.tb04504.x?_sg%5B0%5D=zK7kCTVwuGsGnJIhW_-UyDNZ3IxTpqhn7LSknneEUPLaAq3bfryJ_67k5qHEvUGKv2_VGSNXLZEMIMrJ78KA3AyfJg.uWHssCRJPjEyAjHX4M8z5vgqIvDTBFdR9WQca0UUi6kkAX0FGQVl5SWme6LDppoClh-Nrjux1bYSHJqTk5Mimw)
4. Blasi, É. A., Buffon, G., Rativa, A. G., Lopes, M. C., Berger, M., Santi, L., et al. (2017). High infestation levels of *Schizotetranychus oryzae* severely affects rice metabolism. *J. Plant Physiol.* 219:100-111. doi:[10.1016/j.jplph.2017.10.005](https://doi.org/10.1016/j.jplph.2017.10.005)
5. Bownes, A., Hill, M. P., and Byrne, M. J. (2010). Evaluating the impact of herbivory by a grasshopper, *Cornops aquaticum* (Orthoptera: Acrididae), on the competitive performance and biomass accumulation of water hyacinth, *Eichhornia crassipes* (Pontederiaceae). *Biol. Control* 53:297-303. doi:[10.1016/j.biocontrol.2010.02.013](https://doi.org/10.1016/j.biocontrol.2010.02.013)
6. Bufon, G., dos Reis Blasi, É. A., Lamb, T. I., Adamski, J. M., Schwambach, J., Ricachenevsky, F. K., et al. (2020). Nipponbare and wild rice species as unexpected tolerance and susceptibility sources against *Schizotetranychus oryzae* (Acari: Tetranychidae) mite infestation. *bioRxiv* 2020.01.22.914184v1; doi:10.1101/2020.01.22.914184
7. Câmara, T., Arnan, X., Barbosa, V. S., Wirth, R., Iannuzzi, L., and Leal, I. R. (2020). Disentangling the effects of foliar vs. floral herbivory of leaf-cutting ants on the plant reproductive success of *Miconia nervosa* (Smith) Triana (Family Melastomataceae). *Bull. Entomol. Res*. 110:77-83. doi:[10.1017/S000748531900029](https://doi.org/10.1017/S0007485319000294)
8. Collins, C. M., Rosado, R. G., and Leather, S. R. (2001). The impact of the aphids *Tuberolachnus salignus* and *Pterocomma salicis* on willow trees. *Ann. Appl. Biol.* 138:133-140. doi:10.1111/j.1744-7348.2001.tb00095.x
9. Delaney, K. J., Haile, F. J., Peterson, R. K. D., and Higley, L. G. (2008). Impairment of leaf photosynthesis after insect herbivory or mechanical injury on common milkweed, *Asclepias syriaca*. *Environ. Entomol.* 37:1332-1343. doi:10.1603/0046-225x(2008)37[1332:iolpai]2.0.co;2
10. Delaney, K. J. (2012). *Nerium oleander* indirect leaf photosynthesis and light harvesting reductions after clipping injury or *Spodoptera eridania* herbivory: high sensitivity to injury*. Plant Sci*. 185:218-226. doi:[10.1016/j.plantsci.2011.10.012](https://doi.org/10.1016/j.plantsci.2011.10.012)
11. Donovan, M. P., Nabity, P. D., and DeLucia, E. H. (2013). Salicylic acid-mediated reductions in yield in *Nicotiana attenuata* challenged by aphid herbivory. *Arthropod-Plant Inte.* *7*:45-52. doi:10.1007/s11829-012-9220-5
12. Gianoli, E., and Niemeyer, H. M. (1997). Lack of costs of herbivory-induced defenses in a wild wheat: integration of physiological and ecological approaches. *Oikos* 80:269-275. doi:10.2307/3546595
13. Gore, J., Cook, D. R., Catchot, A. L., Musser, F. R., Stewart, S. D., Leonard, B. R., et al. (2013). Impact of twospotted spider mite (Acari: Tetranychidae) infestation timing on cotton yields. *J. Cotton Sci.* 17:34-39.
14. Haile, F. J., Higley, L. G., Ni, X., and Quisenberry, S. S. (1999). Physiological and growth tolerance in wheat to Russian wheat aphid (Homoptera: Aphididae) injury. *Environ. Entomol*. 28:787-794. doi:[10.1093/ee/28.5.787](https://www.researchgate.net/deref/http%3A%2F%2Fdx.doi.org%2F10.1093%2Fee%2F28.5.787?_sg%5B0%5D=0RSyrIeysyp1cv9sxGVCiVwZanoLIysZ2EfcKWXdpec6t8eM6JnXIL1fq3Y0UVB8tUW5LQwS6-k6XOH_hsyTHx529g.je7_rVYYu6k9uKRZcIuhZIkzeR4t0ySiNkGjGPH8uCYA1SBOs-27OHwDLhA9fs4tM94RF1kzNCDptCng2Aw8aQ)
15. Haile, F. J., and Higley, L. G. (2003). Changes in soybean gas-exchange after moisture stress and spider mite injury. *Environ. Entomol.* 32:433-440. doi:[10.1603/0046-225X-32.3.433](https://www.researchgate.net/deref/http%3A%2F%2Fdx.doi.org%2F10.1603%2F0046-225X-32.3.433?_sg%5B0%5D=XIGs92_8wKc4kUFubK0kD8HrH4JhjYhzsg1GuXPQbOoBmCtmR3CIbQ3LxBPaDnQGXc_tlK58ypTMj0TWLoyZRbf9tg.M_iXsFCfszuMDsfTy-oziTs7RviKBXLeFiE2KPAT4wr1hB9g6vnm5vrabs8MStCQZE3Vs9Bc3KJqpVEfGT3LuA)
16. Halitschke, R., Hamilton, J. G., and Kessler, A. (2011). Herbivore‐specific elicitation of photosynthesis by mirid bug salivary secretions in the wild tobacco *Nicotiana attenuata*. *New Phytol*. 191:528-535. doi:10.1111/j.1469-8137.2011.03701.x
17. Harper, A. M., and Kaldy, M. S. (1982). Effect of the pea aphid, *Acyrthosiphon pisum* (Hemiptera (Homoptera): Aphididae), on yield and quality of forage alfalfa. *Can Entomol*. 114:485-489. doi:[10.4039/Ent114485-6](https://doi.org/10.4039/Ent114485-6)
18. Heng-Moss, T., Macedo, T., Franzen, L., Baxendale, F., Higley, L., and Sarath, G. (2006). Physiological responses of resistant and susceptible buffalograsses to *Blissus occiduus* (Hemiptera: Blissidae) feeding. *J. Econ. Entomol*. 99:222-228. doi:[10.1603/0022-0493(2006)099[0222:proras]2.0.co;2](https://doi.org/10.1603/0022-0493(2006)099%5B0222:proras%5D2.0.co;2)
19. Hutchison, W. D., and Campbell, C. D. (1994). Economic impact of sugarbeet root aphid (Homoptera: Aphididae) on sugarbeet yield and quality in southern Minnesota. *J. Econ. Entomol*. 87:465-475. doi:[10.1093/jee/87.2.465](https://doi.org/10.1093/jee/87.2.465)
20. Jing, J., Raaijmakers, C., Kostenko, O., Kos, M., Mulder, P. P., and Bezemer, T. M. (2015). Interactive effects of above-and belowground herbivory and plant competition on plant growth and defence. *Basic Appl Ecol*. 16:500-509. doi:[10.1016/j.baae.2015.04.009](https://doi.org/10.1016/j.baae.2015.04.009)
21. Kessler, A., and T. Baldwin, I. (2004). Herbivore‐induced plant vaccination. Part I. The orchestration of plant defenses in nature and their fitness consequences in the wild tobacco *Nicotiana attenuata*. *Plant J.* 38:639-649. doi:[10.1111/j.1365-313X.2004.02076.x](https://doi.org/10.1111/j.1365-313x.2004.02076.x)
22. Kleinjan, C. A., Edwards, P. B., and Hoffmann, J. H. (2004). Impact of foliage feeding by *Zygina* sp. on tuber biomass and reproduction of *Asparagus asparagoides* (L.): relevance to biological control in Australia. *Biol Control* 30:36-41. doi:[10.1016/j.biocontrol.2003.09.012](https://doi.org/10.1016/j.biocontrol.2003.09.012)
23. Kozel, A. V., Zvereva, E. L., and Kozlov, M. V. (2017). Impacts of root herbivory on seedlings of three species of boreal forest trees. *Appl. Soil Ecol.* 117:203-207. doi:[10.1016/j.apsoil.2017.05.011](https://doi.org/10.1016/j.apsoil.2017.05.011)
24. Lamp, W. O., Nielsen, G. R., Fuentes, C. B., and Quebedeaux, B. (2004). Feeding site preference of potato leafhopper (Homoptera: Cicadellidae) on alfalfa and its effect on photosynthesis. *J. Agric. Urban Entomol.* 21:25-38.
25. Liu, J., Legarrea, S., and Kant, M. R. (2017). Tomato reproductive success is equally affected by herbivores that induce or that suppress defenses. *Front. Plant Sci.* 8:2128. doi:10.3389/fpls.2017.02128
26. Macedo, T. B., Bastos, C. S., Higley, L. G., Ostlie, K. R., and Madhavan, S. (2003). Photosynthetic responses of soybean to soybean aphid (Homoptera: Aphididae) injury*. J. Econ. Entomol*. 96:188-193. doi:[10.1603/0022-0493-96.1.188](https://www.researchgate.net/deref/http%3A%2F%2Fdx.doi.org%2F10.1603%2F0022-0493-96.1.188?_sg%5B0%5D=WPK1lTJ8ZV1clKOiEyKz_i6-Ki3LtYsMR0KPIlx2CNPqZp-QyXIhqjax8e14C5eaCoKkgCsorgZGBi7O30wQTjg0FA.q4nNhei1nEREN4KrFHqTuYkhtT-gli7qBZ4QVSRC53QYsfj_jBILGnI3PC5Tys1HatLNrc1XniG0Ql-GHCezWw)
27. Macedo, T. B., Peterson, R. K., Weaver, D. K., and Ni, X. (2009). Impact of *Diuraphis noxia* and *Rhopalosiphum padi* (Hemiptera: Aphididae) on primary physiology of four near-isogenic wheat lines. *J. Econ. Entomol*. 102:412-421. doi:[10.1603/029.102.0154](https://www.researchgate.net/deref/http%3A%2F%2Fdx.doi.org%2F10.1603%2F029.102.0154?_sg%5B0%5D=jRPW5nH7lHo7yAxGdR7TWT8H2Mdb1R3-NaxfQ1YnVkYDDhhu31wQu7qL4x8-mgJiF9znwfWyDxv47iW3cOrhr7ggew.aR8lU33R9268xfNXWAA6ApmC_tDkYvlm7XgtaVBMI9lKmZquy9jDY4Gb3cNNTxNLSD1Y8mc_eXVr25WPDMqYxA)
28. Mundim, F. M., Alborn, H. T., Vieira‐Neto, E. H., and Bruna, E. M. (2017). A whole‐plant perspective reveals unexpected impacts of above‐and belowground herbivores on plant growth and defense. *Ecology* 98:70-78. doi:10.1002/ecy.1619
29. Park, Y. L., and Lee, J. H. (2005). Impact of twospotted spider mite (Acari: Tetranychidae) on growth and productivity of glasshouse cucumbers. *J. Econ. Entomol.* 98:457-463. doi:10.1093/jee/98.2.457
30. Peschiutta, M. L., Scholz, F. G., Goldstein, G., and Bucci, S. J. (2020). Lagged effects of sawfly leaf herbivory on reproductive organs in cherry trees: Overcompensation in flower production reduces quality of fruits and seeds. *Basic Appl Ecol*. 45:22-30. doi:[10.1016/j.baae.2020.03.006](https://doi.org/10.1016/j.baae.2020.03.006)
31. Pincebourde, S., Frak, E. L. A., Sinoquet, H., Regnard, J. L., and Casas, J. (2006). Herbivory mitigation through increased water‐use efficiency in a leaf‐mining moth–apple tree relationship. *Plant Cell Environ.* 29:2238-2247. doi:[10.1111/j.1365-3040.2006.01598.x](https://doi.org/10.1111/j.1365-3040.2006.01598.x)
32. Poveda, K., Steffan-Dewenter, I., Scheu, S., and Tscharntke, T. (2003). Effects of below-and above-ground herbivores on plant growth, flower visitation and seed set. *Oecologia* 135:601-605. doi:10.1007/s00442-003-1228-1
33. Preisser, E. L., Gibson, S. E., Adler, L. S., and Lewis, E. E. (2007). Underground herbivory and the costs of constitutive defense in tobacco. *Acta Oecol.* 31:210-215. doi:10.1016/j.actao.2006.09.004
34. Reddall, A., Sadras, V. O., Wilson, L. J., and Gregg, P. C. (2004). Physiological responses of cotton to two‐spotted spider mite damage. *Crop Sci.* 44:835-846. doi:[10.2135/cropsci2004.8350](https://www.researchgate.net/deref/http%3A%2F%2Fdx.doi.org%2F10.2135%2Fcropsci2004.8350?_sg%5B0%5D=a7jd8KyUP1Uq_iK6e1Txxaf5_wJ7hmJV_E0zD-Ve2zb_3N07yVz1M-g28b-CZD-ExTkoHau4M287y8c9swhblrMfTg.iUnYyeJVo2-_qK8C6oM7bMqRdTkfn4C7ck2DtJ35lY1z3up2jB6QsDwhA6DIQ3MpG3qdCPln9OugInjcD00vZA)
35. Retuerto, R., Fernandez‐Lema, B., and Obeso, J. R. (2004). Increased photosynthetic performance in holly trees infested by scale insects. *Funct. Ecol.* 18:664-669. doi:[10.1111/j.0269-8463.2004.00889.x](https://www.researchgate.net/deref/http%3A%2F%2Fdx.doi.org%2F10.1111%2Fj.0269-8463.2004.00889.x?_sg%5B0%5D=-kIfFQ4kSqIceT1JmF-5l-0gzw8yYP2AdrGSn5AHnva5BF5ffxAXnIkcFjq2QI5lFwCR-y7Zgt1FtphJNm_q3a3pwQ.0RPnQccvZ1c1Pfthi-ec2GydYfLDpUR6jhz8wkDBrtWclg1Iy15sfyHoYOc_Ko6NIamNOXXme6Gj0aTj4N6JMg)
36. Roubíčková, A., Mudrák, O., and Frouz, J. (2012). The effect of belowground herbivory by wireworms (Coleoptera: Elateridae) on performance of *Calamagrostis epigejos* (L) Roth in post-mining sites. *Eur. J. Soil Biol.* 50:51-55. doi:[10.1016/j.ejsobi.2011.12.004](https://doi.org/10.1016/j.ejsobi.2011.12.004)
37. Sanford, L. L., and Webb, R. E. (1977). Loss of yield in potato selections infested with potato leafhoppers. *Am. Potato J*. 54:581-586. doi:10.1007/BF02855285
38. Santamaria, M. E., Martinez, M., Arnaiz, A., Ortego, F., Grbic, V., and Diaz, I. (2017). MATI, a novel protein involved in the regulation of herbivore-associated signaling pathways. *Front. Plant Sci.* 8:975. doi:10.3389/fpls.2017.00975
39. Santamaria, M. E., Diaz, I., and Martinez, M. (2018). Dehydration stress contributes to the enhancement of plant defense response and mite performance on barley. *Front. Plant Sci.* 9:458. doi:[10.3389/fpls.2018.00458](https://doi.org/10.3389/fpls.2018.00458)
40. Schmidt, L., Schurr, U., and Roese, U. S. (2009). Local and systemic effects of two herbivores with different feeding mechanisms on primary metabolism of cotton leaves*. Plant Cell Environ.* 32:893-903. doi:[10.1111/j.1365-3040.2009.01969.x](https://doi.org/10.1111/j.1365-3040.2009.01969.x)
41. Sotelo, P., Pérez, E., Najar-Rodriguez, A., Walter, A., and Dorn, S. (2014). Brassica plant responses to mild herbivore stress elicited by two specialist insects from different feeding guilds. *J. Chem. Ecol.* 40:136-149. doi:[10.1007/s10886-014-0386-4](https://doi.org/10.1007/s10886-014-0386-4)
42. Tang, J. Y., Zielinski, R. E., Zangerl, A. R., Crofts, A. R., Berenbaum, M. R., and DeLucia, E. H. (2006). The differential effects of herbivory by first and fourth instars of *Trichoplusia ni* (Lepidoptera: Noctuidae) on photosynthesis in *Arabidopsis thaliana*. *J. Exp. Bot.* 57:527-536. doi:[10.1093/jxb/erj032](https://doi.org/10.1093/jxb/erj032)
43. Velikova, V., Salerno, G., Frati, F., Peri, E., Conti, E., Colazza, S., & Loreto, F. (2010). Influence of feeding and oviposition by phytophagous pentatomids on photosynthesis of herbaceous plants. *J. Chem. Ecol.* 36:629-641. doi:[10.1007/s10886-010-9801-7](https://doi.org/10.1007/s10886-010-9801-7)
44. Vranjic, J. A., and Ash, J. E. (1997). Scale insects consistently affect roots more than shoots: the impact of infestation size on growth of eucalypt seedlings. *J. Ecol.* 85:143-149. doi:10.2307/2960646
45. Watanabe, T., and Kitagawa, H. (2000). Photosynthesis and translocation of assimilates in rice plants following phloem feeding by the planthopper *Nilaparvata lugens* (Homoptera: Delphacidae). *J. Econ. Entomol.* 93:1192-1198. doi:[10.1603/0022-0493-93.4.1192](https://doi.org/10.1603/0022-0493-93.4.1192)
46. Zangerl, A. R., Arntz, A. M., and Berenbaum, M. R. (1997). Physiological price of an induced chemical defense: photosynthesis, respiration, biosynthesis, and growth. *Oecologia* 109:433-441. doi:10.1007/s004420050103

**Supplementary Table 2.** Publication bias risk assessment. Fail-safe numbers obtained using the Rosenberg approach (α 0.05) and Egger’s test p-values for each analysis. Fail-safe numbers greater than 5N+10 indicate that the data are robust. N = number of experiments included in the meta-analysis. P-values higher than 0.05 suggest that no publication bias are present in the data. When trim and fill procedure was applied, fail-safe number and Egger’s test p-values shown represent the values obtained after the procedure was used. An asterisk indicates robust data rejecting publication bias.

| **Parameter** | | | **Fail-safe number** | **N** | **Egger test (p-value)** | **Trim and fill procedure** |
| --- | --- | --- | --- | --- | --- | --- |
| **Growth** | | | 1105* | 62 | 0.00023 | No |
|  | **Variables** | |  |  |  |  |
|  | Feeding guilt | |  |  |  |  |
|  |  | Phloem-feeding | 227* | 16 | 0.02763 | No |
|  |  | Chewing | 7 | 17 | 0.20972* | No |
|  |  | Cell-content feeder | 153* | 11 | 0.83805* | No |
|  |  | Gall-forming | 0 | 7 | 0.76674* | Yes |
|  | Plant host | |  |  |  |  |
|  |  | Crop | 175* | 19 | 0.42328* | No |
|  |  | Herbaceous | 8 | 18 | 0.11396* | No |
|  |  | Woody | 26 | 10 | 0.62628* | Yes |
|  | Plant stage | |  |  |  |  |
|  |  | Early vegetative stage | 294* | 28 | 0.0762* | No |
|  |  | Late vegetative stage | 85* | 10 | 0.17195* | No |
|  |  | Reproductive stage | 0 | 9 | 0.37161* | No |
|  | Infestation lenght | |  |  |  |  |
|  |  | Short-term | 0 | 10 | 0.95847* | No |
|  |  | Long-term | 466* | 33 | 0.00113 | No |
|  | Infestation magnitude | |  |  |  |  |
|  |  | Light | 0 | 15 | 0.86523* | No |
|  |  | Medium | 47 | 19 | 0.42526* | No |
|  |  | Heavy | 235* | 11 | 0.01249 | No |
|  | | | | | | |
| **Photosynthesis** | | | 2587* | 88 | 0.00005 | No |
|  | **Variables** | |  |  |  |  |
|  | Feeding guilt | |  |  |  |  |
|  |  | Phloem-feeding | 197* | 28 | 0.00086 | No |
|  |  | Chewing | 29 | 14 | 0.11082* | No |
|  |  | Cell-content feeder | 345* | 20 | 0.00005 | No |
|  |  | Gall-forming | 0 | 5 | 0.00271 | Yes |
|  |  | Leafminer | 44* | 3 | 0.18925* | No |
|  | Plant host | |  |  |  |  |
|  |  | Crop | 618* | 49 | 0.0003 | No |
|  |  | Herbaceous | 110* | 9 | 0.03665 | No |
|  |  | Woody | 0 | 11 | 0.08038* | No |
|  | Plant stage | |  |  |  |  |
|  |  | Early vegetative stage | 464* | 29 | 0.00287 | No |
|  |  | Late vegetative stage | 156* | 23 | 0.00132 | No |
|  |  | Reproductive stage | 7 | 6 | 0.8468* | Yes |
|  | Infestation lenght | |  |  |  |  |
|  |  | Short-term | 448* | 39 | 0.04352 | No |
|  |  | Long-term | 487* | 23 | 0.00044 | No |
|  | Infestation magnitude | |  |  |  |  |
|  |  | Light | 0 | 33 | 0.85929* | No |
|  |  | Medium | 352* | 17 | 0.00212 | No |
|  |  | Heavy | 162* | 18 | 0.00323 | No |
|  | | | | | | |
| **Reproduction** | | | 449* | 42 | 0.00417 | No |
|  | **Variables** | |  |  |  |  |
|  | Feeding guilt | |  |  |  |  |
|  |  | Phloem-feeding | 5 | 3 | 0.76814* | Yes |
|  |  | Chewing | 67 | 14 | 0.84094* | No |
|  |  | Cell-content feeder | 91* | 11 | 0.00241 | No |
|  | Plant host | |  |  |  |  |
|  |  | Crop | 112* | 15 | 0.01226 | No |
|  |  | Herbaceous | 33 | 8 | 0.68816* | Yes |
|  |  | Woody | 6 | 7 | 0.34419* | Yes |
|  | Plant stage | |  |  |  |  |
|  |  | Early vegetative stage | 65 | 13 | 0.59653* | No |
|  |  | Late vegetative stage | 55 | 16 | 0.93976* | No |
|  | Infestation lenght | |  |  |  |  |
|  |  | Short-term | 9 | 4 | 0.9379* | Yes |
|  |  | Long-term | 195* | 22 | 0.22279* | No |
|  | Infestation magnitude | |  |  |  |  |
|  |  | Light | 0 | 6 | 0.00607 | Yes |
|  |  | Medium | 120* | 17 | 0.16589* | No |
|  |  | Heavy | 94* | 11 | 0.04939 | No |
|  | | | | | | |
| **Carbohydrate content** | | | 0 | 17 | 0.64227* | No |
